# S Trimer Derived from SARS-CoV-2 B.1.351 and B.1.618 Induced Effective Immune Response against Multiple SARS-CoV-2 Variants

**DOI:** 10.3390/vaccines11010193

**Published:** 2023-01-16

**Authors:** Hongye Wang, Zengshuai Wang, Liang Ma, Xiaoyong Zhu, Bingxiang Li, Yuhang Huang, Jingwen Li, Ming Sun, Li Shi, Yufeng Yao

**Affiliations:** 1 Institute of Medical Biology, Chinese Academy of Medical Sciences & Peking Union Medical College, Kunming 650118, China; 2 School of Basic Medical Science, Kunming Medical University, Kunming 650500, China; 3 School of Life Sciences, Yunnan University, Kunming 650091, China

**Keywords:** SARS-CoV-2, spike, trimer, beta variant, immune response

## Abstract

The spread of SARS-CoV-2 and its variants leads to a heavy burden on healthcare and the global economy, highlighting the need for developing vaccines that induce broad immunity against coronavirus. Here, we explored the immunogenicity of monovalent or bivalent spike (S) trimer subunit vaccines derived from SARS-CoV-2 B.1.351 (S1-2P) or/and B.1. 618 (S2-2P) in Balb/c mice. Both S1-2P and S2-2P elicited anti-spike antibody responses, and alum adjuvant induced higher levels of antibodies than Addavax adjuvant. The dose responses of the vaccines on immunogenicity were evaluated in vivo. A low dose of 5 μg monovalent recombinant protein or 2.5 μg bivalent vaccine triggered high-titer antibodies that showed cross-activity to Beta, Delta, and Gamma RBD in mice. The third immunization dose could boost (1.1 to 40.6 times) high levels of cross-binding antibodies and elicit high titers of neutralizing antibodies (64 to 1024) prototype, Beta, Delta, and Omicron variants. Furthermore, the vaccines were able to provoke a Th1-biased cellular immune response. Significantly, at the same antigen dose, S1-2P immune sera induced stronger broadly neutralizing antibodies against prototype, Beta, Delta, and Omicron variants compared to that induced by S2-2P. At the same time, the low dose of bivalent vaccine containing S2-2P and S1-2P (2.5 μg for each antigen) significantly improved the cross-neutralizing antibody responses. In conclusion, our results showed that monovalent S1-2P subunit vaccine or bivalent vaccine (S1-2P and S2-2P) induced potent humoral and cellular responses against multiple SARS-CoV-2 variants and provided valuable information for the development of recombinant protein-based SARS-CoV-2 vaccines that protect against emerging SARS-CoV-2 variants.

## 1. Introduction

The Coronavirus disease 2019 (COVID-19) pandemic caused by severe acute respiratory syndrome coronavirus 2 (SARS-CoV-2) has led to a heavy burden on the global healthcare system and significant economic losses [1]. Developing a safe, effective, and long-lasting SARS-CoV-2 vaccine is a common aspiration worldwide. Multiple vaccines based on the prototype strain of SASR-CoV-2 have been approved and have been protecting vaccinated people against severe disease and infection [2]. However, vaccine effectiveness declines, and protection against emerging mutants causing new outbreaks in various countries and regions has waned over time [3,4,5,6]. Throughout the pandemic, more than 1000 SARS-CoV-2 variants have been reported, five of which include B.1.1.7 (Alpha), B.1.351 (Beta), P.1 (Gamma), B.1.617.2 (Delta), and B.1.521 (Omicron) have been identified as variants of concern (VOCs). Except for alpha variants, these variants show substantially decreased neutralization by the existing monoclonal antibodies and sera from vaccinators and individuals who recovered from the first wave of the pandemic [7,8,9,10,11,12]. Thus, a more effective and broad-spectrum protective vaccine against SARS-CoV-2 is urgently required.

The trimeric spike glycoprotein (S) is the major surface protein of SARS-CoV-2 that consists of the S1 subunit and S2 subunit. In the course of infection, the S protein initiates major viral entry via binding the receptor-binding domain (RBD) on the S1 subunit to the host receptor angiotensin-converting enzyme 2 (ACE-2) which is the principal target of eliciting neutralizing responses [13]. S-specific IgG, especially RBD-specific IgG elicited during viral infection or after vaccination, positively correlated with serum-neutralizing antibody titers [14,15]. The spike protein is a crucial antigen for the rational design of vaccines inducing neutralizing antibodies. Different mutations within the spike protein were frequently observed, and the antigenicity and immunogenicity of different mutants vary [8,16,17]. Various studies reported that mutations in the spike protein of the Beta variant (mainly on NTD and RBD) considerably reduced vaccine efficacy and neutralizing sensitivity [8]. Approximal 11-33-fold reductions in serum sensitivity to convalescent sera and a 3.4-8.5-fold reduction in sera sensitivity to vaccinators were observed against the beta variant [18,19,20]. In contrast, infection of the Beta variant elicited high titers of spike-binding and neutralizing antibodies against both the prototype and gamma variant, indicating Beta spike protein as a promising candidate for inducing cross-reactive neutralizing antibody responses to SARS-CoV-2 [21]. In addition, the Delta variant, Kappa variant (B.1.617.1), and B.1.618 variant exhibited a significantly increased binding affinity with nonhuman ACE2 orthologs than prototype spike but reduced neutralizing sensitivity to convalescent sera [22]. B.1.618 possesses two deletions of Tyr145 and His146 at the NTD and an E484K mutation at the RBD, causing antibody escape due to high ACE2 affinity [23].

Strategies for using trimer subunit antigens against SARS-CoV-2 have been explored to confront the emergence of mutants. Previous studies showed subunit vaccine using the prefusion-stabilized prototype SARS-CoV-2 S trimer produced from ExpiCHO-s cells (transfected with codon-optimized gene encoding SARS-CoV-2 S ectodomain with mutated furin-recognition site, K986P-V986P mutations, and C-terminal T4 foldon) adjuvanted with CpG 1018 and alum showed effective protection in mice and nonhuman primates [24] and exhibited safety and efficacy in clinical trials [25]. Similarly, trimer antigen that contains trimer-tag at the C terminal of spike ectodomain adjuvanted with either AS03 or CpG 1018 plus alum adjuvants induced high levels of both humoral and cell-mediated immune responses and provided protection in rodents and nonhuman primates [26], and showed good safety and promising protective efficacy in clinical trials [27,28]. Notably, the production of spike trimer that contains ectodomain of spike and T4 foldon using CHO expression system was reported to have achieved high yield and good quality that maintained trimer configuration [29]. Since newly emerging variants with higher immune escape ability might challenge the protective immune barrier established by existing vaccines, it is worth exploring the immunogenicity and protective efficacy of the recombinant subunit vaccine based on the spike proteins of emerging SARS-CoV-2 variants. 

Here, to evaluate the immunogenicity of variant-specific spike subunit vaccine in broad-spectrum neutralizing capacity, we designed a monovalent S trimer subunit vaccine derived from spike protein B.1.351 or B.1.1.628 and a bivalent vaccine based on the combination of the two spike glycoproteins. The spike was stabilized in its trimer conformation by two substitutions of proline mutations and a T4 foldon on the C-terminal and adjuvanted with alum or a squalene-based oil-in-water emulsion similar to MF59^®^ named AddaVax. The immunogenicity of two monovalent vaccines and the bivalent vaccine at different doses was evaluated in BALB/c mice. The cross-binding antibodies against different S and RBD antigens were detected by coating various proteins in the enzyme-linked immunosorbent (ELISA) assay, and the cross-neutralizing antibodies against the SARS-CoV-2 prototype, Beta, Delta, and Omicron strains from different lineages were identified. Splenocytes harvested after the third vaccination were used for the detection of Th1 (IL-2 and IFNγ) and Th2 (IL-4 and IL-5) cellular immune responses with the enzyme-linked immunospot (ELISPOT) assay. Our findings provide valuable information for developing recombinant protein-based SARS-CoV-2 vaccines and a good foundation for booster vaccinations to protect against emerging SARS-CoV-2 variants.

## 2. Materials and methods

### 2.1. Cells, Plasmids, and Viruses

HEK293T, BHK21, and Vero-E6 cells were cultured in Dulbecco’s modified Eagle’s medium (DMEM; Gibco, CN) supplemented with 10% fetal bovine serum (FBS; Gibco, AU) and 0.1% penicillin and streptomycin. 293T-ACE2 cells that overexpression of human ACE2 were cultured in DMEM supplemented with 10% FBS and 50 μg/mL G418. CHO-S cells were cultured in Forti-CD CHO medium (Gibco) supplemented with 3% glutamic acid, and the vector for env expression was pcDNA™3.1 (Invitrogen, CN). A plasmid corresponding to the semi-stabilized SARS-CoV-2 B.1.351 S1-2P (residues 16-1213, with K986P and V987P mutation) or B.1.618 S2-2P (EPI_ISL_4363578, residues 16-1213, with K986P and V987P mutation) with tissue plasminogen activator (tPA) signal peptide protein at N terminal and T4 trimerization sequence and 6X His at C terminal was codon-optimized and synthesized by Tsingke Biotechnology Co., Ltd. The plasmids were then cloned into a gWiz-blank vector under the control of CMV promoter with intron A. SARS-CoV-2 prototype, Beta variant, Delta variant (CCPM-B-V049-2105-8), and Omicron variant (BA.1 like, which contains more than 30 mutations on spike, including A67V, D614G, D796Y, E484A, G142D, G339D, G446S, G496S, H69del, H655Y, ins214EPE, K417N, L212I, L981F, N211del, N440K, N501Y, N679K, N764K, N856K, N969K, P681H, Q493R, Q498R, Q954H, S371L, S373P, S375F, S477N, T95I, T478K, T547K, V70del, V143del, Y144del, Y145del, and Y505H) were propagated in Vero E6 cells and titrated by TCID50 assay on Vero E6 cells at the Institute of Medical Biology, Chinese Academy of Medical Sciences (IMBCAMS).

### 2.2. Proteins, Expression, and Purification

The expression vector of the S trimer was transfected into the CHO-S cell line according to the manufacturer’s instructions. Briefly, CHO-S cells were seeded at 6 × 10^5^ cells/mL in Forti-CD CHO medium (Gibco, Shanghai, China, A11483) and incubated in an orbital shaking incubator at 37 °C and 120 rpm with 8% CO_2_ overnight. The next day, 1.0 × 10^6^ cells/mL were transfected using FreeStyleMax transfection reagent (Thermo Fisher, Waltham, MA, USA, 16447100) and grown for five days at 35 °C. The cell culture medium was harvested by centrifuge at 4800 rpm to remove cells. The supernatant was collected and passed through a 0.45 μm filter and then loaded onto the pre-equilibrated Ni Smart Beads 6FF (Changzhou Smart-Lifesciences Biotechnology Co., Ltd., Changzhou, China, SA036100) affinity column for protein purification. The eluted proteins were then buffer exchanged to PBS by ultracentrifugation using 50 kDa ultrafiltration cubes (Cytiva, Shanghai, China, 28932362).

The S1-D614G, Omicron S trimer, E484K RBD, N501Y RBD, Beta RBD, Delta RBD, and Gamma RBD proteins used in this study were purchased from Sino Biological (Beijing, China) or Novoprotein (Shanghai, China), and detailed information is listed in Supplementary Table S1.

### 2.3. Protein Electrophoresis and Western Blot

For Sodium dodecyl sulfate-polyacrylamide gel electrophoresis (SDS-PAGE), the recombinant S protein was mixed with 5× SDS-PAGE loading buffer (Beyotime Biotechnology, Shanghai, China, P0015L), boiled for 5 min, and briefly centrifuged to collect water droplets on the upper cover. Next, the proteins were separated by 10% SDS-PAGE. For Native-PAGE, the recombinant S protein was mixed with a 2× PAGE loading buffer and separated by 8% Bis–Tris Protein Gels. For Coomassie Bright Blue staining, CBB Fast Staining Solution (Tiangen Biotech, Beijing, China) was used. For Western blot (WB) analysis, the proteins were transferred from the gel to a PVDF membrane after blocking by 5% milk, three washes in PBS with 0.1% Tween 20 (PBST), the membranes were incubated with the HRP-conjugated goat anti 6× His antibody for one hour and washed three times in PBST. Then, the protein level was visualized by enhanced chemiluminescence (ECL) (Bio-Rad, Hercules, CA, USA).

### 2.4. Animal Vaccination 

Female BALB/c mice 6–8 weeks old were supplied by the Central Services of the Institute of Medical Biology, Chinese Academy of Medical Sciences & Peking Union Medical College (IMB-CAMS). Mice were divided into different groups, with four mice in each group. Recombinant S proteins were diluted in 25 μL of phosphate-buffered saline (PBS) and mixed with the same volume of alum adjuvant or squalene-based oil-in-water emulsion adjuvant AddaVax (InvivoGen, San Diego, CA, USA) and immunized intramuscularly at doses of 5–20 μg SARS-CoV-2 S1-2P or S2-2P or both, at weeks 0 and 3, or boosted again at week 6. The blood samples were collected from the caudal vein at two weeks following boost immunization or from orbital veins on the day of sacrifice, followed by centrifugation at 3500× *g* for 10 min to collect serum and stored at −20 °C before use. 

### 2.5. Enzyme-Linked Immunosorbent Assay (ELISA) 

Recombinant SARS-CoV-2 proteins (S1-2P, S2-2P, S1-D614G, Omicron S trimer, E484K RBD, N501Y RBD, Beta RBD, Delta RBD, Gamma RBD) were coated into a 96-well plate at 1 µg/mL in phosphate-buffered saline (PBS) and incubated overnight at 4 °C. The next day, plates were washed three times by PBST (PBS with 0.05% Tween 20), then blocked by 200 μL PBST containing 5% BSA for 2 h at 37 °C. Plates were then washed and incubated with serially diluted serum samples (starting from 100, 3-fold, or 5-fold, 8×) and incubated for 1 h at 37 °C followed by five washes. Total SARS-CoV-2 S (RBD, S1, and S)-specific mouse IgG antibodies were detected using HRP-conjugated anti-mouse (1:10,000) (Theromofisher; Waltham, MA, USA) for 1 h at RT. The plates were washed and developed using TMB (2-Component Microwell Peroxidase Substrate Kit), and the reaction was stopped using 1N phosphoric acid solution. Plates were read at 450 nm wavelength within 20 min using a plate reader (Molecular Devices, San Jose, CA, USA). Absorbance at 620 nm wavelength was also measured to remove background reading. Antibody titers were defined as the reciprocal serum dilution that yielded half-maximal absorbance values.

### 2.6. Enzyme Linked Immunospot Assay (ELISPOT)

Mouse IFN-γ precoated ELISPOT kit (DAKEWEI, Inc., Shenzhen, China, Cat#: 2210001), Mouse IL-4 precoated ELISPOT kit (DAKEWEI, Inc., Cat#: 2210401), Mouse IL-2 ELISpot kit (Mabtech, Inc., Nacka Strand, Sweden, Cat#: 2210401), and Mouse IL-5 ELISpot kit (Mabtech, Inc., Cat#: 2210401) were used in this study. The performances accorded with the manufacturer’s instructions. Briefly, the precoated plates were washed and activated before use, splenocytes of immunized mice were harvested and suspended in RPMI 1640, plated in 96-well plates (2 × 10^5^ cells/well), and cultured with 10 µg/mL of S1-2P and S2-2P recombinant proteins corresponding to the immunogen at 37 °C and 5% CO_2_ for 24 h. The supernatants were discarded, and cells were lysed in ddH_2_O at 4 °C for 10 min (for IFN-γ and IL-4). After washing, biotinylated antibodies were added into wells and incubated at 37 °C for an additional 1–2 h. After 6 washes, streptavidin-HRP (1:100) or streptavidin-ALP (1:1000) was added to the wells. After incubation at 37 °C for 1 h, substrate solution was added to develop blots at 37 °C for 30 min in the darkness. The reaction was stopped by extensively washing in tap water, and wells were air-dried. The plates were inspected and counted using an ELISPOT Reader (Bio Reader 4000 Pro-X, Bio-Sys GmbH, Karben, Germany).

### 2.7. SARS-CoV-2 Neutralization Assay

Neutralizing antibody activities induced by vaccines were titrated based on the inhibition of cytopathogenic effect (CPE). Briefly, 100 μL Vero E6 cells (5 × 10^4^ cells/mL) were placed in the wells of 96 cell plates and cultured for 24 h. An equal volume of serially diluted serum and 100 TCID50 SARS-CoV-2 was mixed and incubated for one hour at 37 °C. Then, the culture medium was discarded, and the mixture of serum and virus was added to Vero cells, followed by incubation at 37 °C for 4 days. CPE was recorded to determine the antibody-neutralizing titer, and the above mixture was added to each well. Subsequently, the cells were cultured for 5 days. An observable CPE was recorded as “+”, and no CPE was recorded as “−”. All these experiments were conducted in a biosafety level 3 laboratory at the Institute of Medical Biology, Chinese Academy of Medical Sciences (IMBCAMS).

### 2.8. Statistical Analysis

All values were presented as mean ± SD. The statistical significance among different vaccination groups was compared using one-way ANOVA or *t*-test using GraphPad Prism 9 software (GraphPad Software Inc., San Diego, CA, USA). Values of *p* < 0.05 were considered significant.

## 3. Results

### 3.1. SARS CoV-2 Spike Expression and Purification

**Figure 1:**
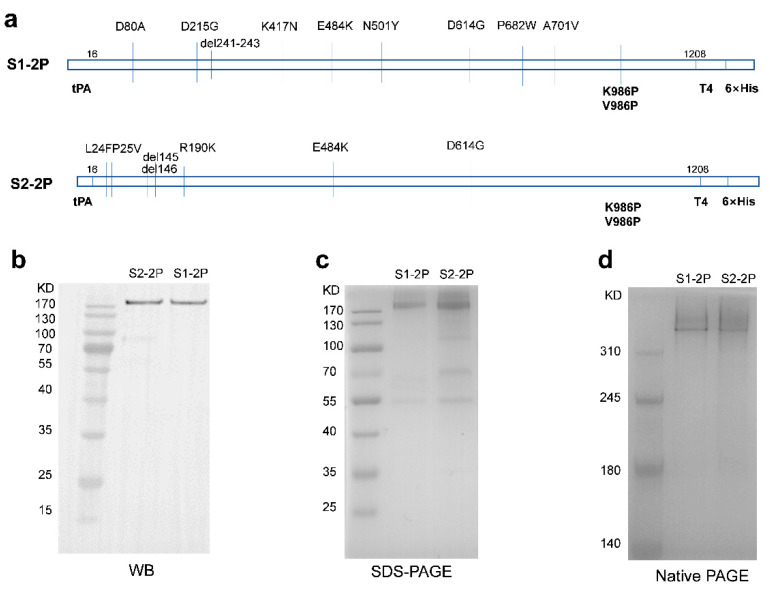
Design, expression, and purification of S-trimer. (**a**) Schematic representations of expression plasmids of SARS-CoV-2 spike protein S1-2P derived from B.1.351 and S2-2P derived from B.1.618. (**b**) WB analysis of purified S protein, HRP-conjugated anti-6× His antibody used as detection antibody. (**c**) Reducing SDS-PAGE analysis with Coomassie Blue staining of purified S protein. (**d**) Native-PAGE analysis with Coomassie Blue staining of purified S protein.

For efficient expression of the ectodomain of SARS CoV-2 spike protein and stabilization of its trimer configuration, the expression plasmid was constructed with residues 16-1208 with tPA signal peptide at N terminal and a T4 foldon and 6× His sequence at C terminal (Figure 1a). The designed S1-2P (derived from B.1.351) and S2-2P (derived from B.1.681) spike plasmids were transiently expressed in CHO-S cells. Five days after transfection, the supernatant was collected, and proteins were purified by His affinity purification and concentrated in PBS solution by ultrafiltration. The production was scaled up from 200-mL shake flasks to 1200-mL shake flasks, and S1-2P and S2-2P yield at about 15 mg/L and 13 mg/L, respectively, in CHO cells. The purified S proteins were detected with HRP-conjugated anti-6× His antibody by Western blot (Figure 1b), indicating the specific antigenicity. An obvious band with a molecular mass of about 180 kDa was observed by reducing SDS-PAGE (Figure 1c). We also evaluated the molecular mass and purity by Native-PAGE. The two T4 foldon expression cassettes correctly produced proteins at the predicted trimer protein molecular weights, higher than 310 kDa (Figure 1d), indicating that the purified S proteins probably showed a classic trimer protein produced by T4 foldon. Meanwhile, reduced and Native-PAGE of the S proteins both achieved good purity.

### 3.2. Immunogenicity of S Trimer in Mice

**Figure 2:**
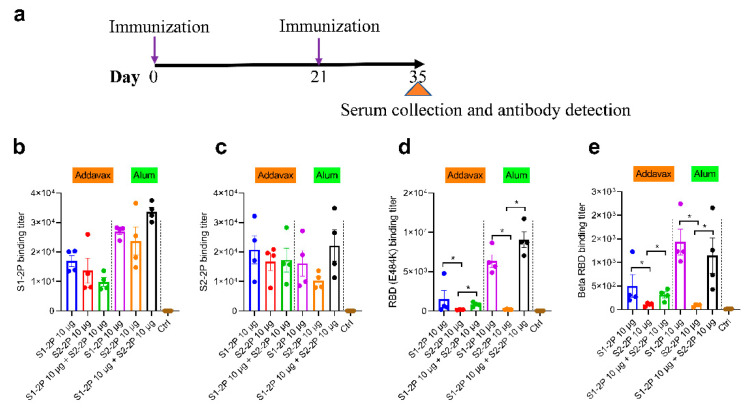
Humoral responses of immunized mice at 10 μg dose. (**a**) Schematic of mouse vaccination. S1-2P-specific IgG titer (**b**), S2-2P-specific IgG titer (**c**), E484K RBD-specific antibody titer (**d**), and Beta RBD-specific antibody titer (**e**) of immunized BALB/c mice were determined by ELISA. The dots of different colors represented mouse serum of different immune groups. The data were represented as the reciprocal of the serum dilution that led to a half-maximal binding signal. Statistical significance was determined using one-way ANOVA or *t*-test and is indicated as * for *p* < 0.05.

To evaluate the immunogenicity of S trimer S1-2P and S2-2P, BABL/c mice were immunized intramuscularly with S1-2P or S2-2P or the combination of S1-2P and S2-2P, either adjuvanted with alum or squalene-based oil-in-water emulsion adjuvant AddaVax. Two injections at a dose of 10 μg were given at an interval of 3 weeks. Blood samples were collected at D35 for antibody detection (Figure 2a). Total IgG antibody titers against the immune antigen S1-2P and S2-2P were determined by ELISA using HRP-conjugated goat anti-mouse IgG antibody. Immunization with S1-2P or S2-2P induced efficient antibody responses against S1-2P and S2-2P, with EC_50_ binding titer reaching 1 × 10^4^ (Figure 2b,c). Groups adjuvanted using alum elicited significantly higher antibody titers than the AddaVax-adjuvanted group. In the AddaVax-adjuvanted group, S1-2P induced better S1-2P-specific antibody response than S2-2P or S1-2P plus S2-2P. In the alum adjuvant group, the group of S1-2P and S2-2P elicited the highest antibody responses, followed by S1-2P and S2-2P. Furthermore, we compared the RBD-specific antibody of the vaccine groups. Similar to the S-specific antibody, the RBD-specific antibody titers were higher in the alum adjuvanted group than AddaVax adjuvanted group. AddaVax-adjuvanted S1-2P elicited higher antibody titer against Beta RBD and E484K RBD than S2-2P or a mixture of S1-2P and S2-2P (*p* < 0.05) (Figure 2d,e). Alum-adjuvanted S1-2P elicited the highest Beta RBD antibody titer (*p* < 0.05), whereas alum-adjuvanted bivalent vaccine elicited the highest E484K antibody titer (*p* < 0.05). Overall, the trimer protein S1-2P or S2-2P showed good immunogenicity, and for the S trimer protein vaccine, alum adjuvant was more efficient in inducing SARS-CoV-2 specific antibody response than AddVax. 

### 3.3. Dose Response of the S Trimer Subunit Vaccine and the Cross-Binding Activity

**Figure 3:**
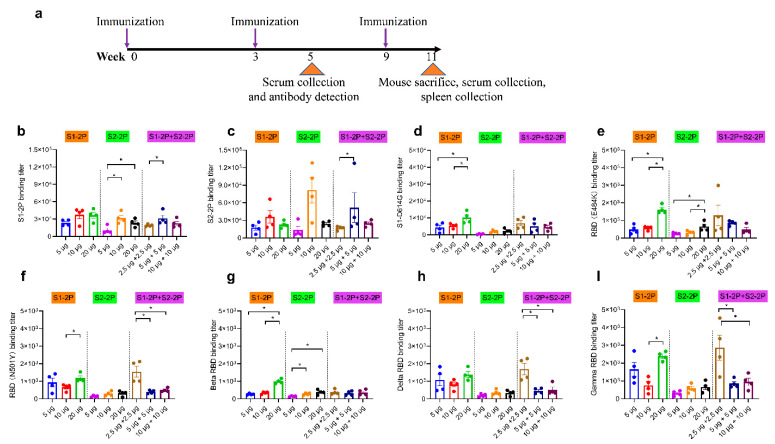
Cross-binding antibodies in the serum of vaccinated mice at different immunization doses. (**a**) Schematic of mouse vaccination. S1-2P-specific IgG titer (**b**), S2-2P-specific IgG titer (**c**), S1-D614G -specific IgG titer (**d**), E484K RBD-specific antibody titer (**e**), N501Y RBD-specific antibody titer (**f**), Beta RBD-specific antibody titer (**g**), Delta RBD-specific antibody titer (**h**), and Gamma RBD-specific antibody titer (**i**) of immunized BALB/c mice were determined by ELISA. The dots of different colors represented mouse serum of different immune groups. The data were represented as the reciprocal of the serum dilution that led to a half-maximal binding signal. Statistical significance was determined using one-way ANOVA or *t*-test and is indicated as * for *p* < 0.05.

To explore the dose response of the S trimer subunit vaccine, 5 μg, 10 μg, and 20 μg of the S trimer monovalent vaccine were used, while 2.5 μg, 5 μg and 10 μg for each antigen were mixed for the bivalent vaccine. Two injections at the designed doses were given in 3-week intervals with alum, as alum was proven more efficient than Addvax. Blood samples were taken at week 5. As a third dose of immunization was administered to improve protective antibody responses in humans, another boost immunization was given in mice at the same dose of each group at week 9, and serum was collected at week 11 (Figure 3a). Total IgG antibody titers against the immune antigens S1-2P and S2-2P were determined by ELISA. A low dose (5 μg) of the S trimer subunit vaccine elicited an efficient antibody response against S1-2P or S2-2P at EC50 titers higher than 10^4^ (Figure 3b,c). The antibody level against S1-2P of 10 μg S1-2P was similar to that of 20 μg S1-2P (EC50 titers > 3 × 10^4^). Immunization with S2-2P also induced S1-2P-targeting antibody, and 10 μg S2-2P or a mixture of 5 μg S1-2P and S2-2P showed the highest S1-2P-binding antibody titers among groups at different doses. Similarly, cross-binding of S2-2P-targeting antibody was also observed in S1-2P immunized groups, and 10 μg S1-2P or bivalent vaccine group with 5 μg S1-2P and S2-2P showed the highest cross-binding titers. Thus, 10 μg of the protein seems to be a good immune dose for the S trimer subunit vaccine (either monovalent or bivalent). The cross-binding activity of antibody response against other S antigens and RBD proteins was further studied. The results showed that antibodies induced by the S trimer subunit vaccine have high antigenic binding ability to a wide spectrum of mutant S or RBD proteins. High titers of S1-D614G antibody were observed in S1-2P immunized group, either alone or in combination with S2-2P, with the highest antibody titer showed in the 20 μg S1-2P immunized group (EC50 titers > 1 × 10^4^) (Figure 3c). The 20 μg S1-2P immunized group also showed high titers of N501Y RBD-, Delta RBD-, Beta RBD, and Gamma RBD-targeting antibodies (Figure 3d–h). By contrast, the cross-binding antibody titers in groups immunized with S2-2P alone were lower than that in the S1-2P group at the same dose. Meanwhile, 2.5 μg S1-2P and 2.5 μg S2-2P led to the highest RBD cross-activity antibody in the bivalent group. The antibody titers against N501Y RBD, Delta RBD, and Gamma RBD were higher than that of the 20 μg S1-2P immunized group. 

**Figure 4:**
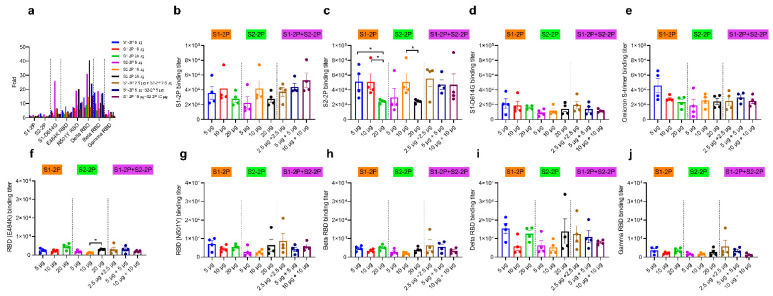
Elevated cross-binding antibody responses after the third immunization. (**a**) The increased fold of serum antibody titer after the third immunization compared with that after the second immunization. S1-2P-specific IgG titer (**b**), S2-2P-specific IgG titer (**c**), S1-D614G -specific IgG titer (**d**), Omicron S trimer-specific IgG titer (**e**), E484K RBD-specific antibody titer (**f**), N501Y RBD-specific antibody titer (**g**), Beta RBD-specific antibody titer (**h**), Delta RBD-specific antibody titer (**i**), and Gamma RBD-specific antibody titer (**j**) of immunized BALB/c mice were determined by ELISA. The dots of different colors represented mouse serum of different immune groups. The data were represented as the reciprocal of the serum dilution that led to a half-maximal binding signal. Statistical significance was determined using one-way ANOVA or *t*-test and is indicated as * for *p* < 0.05.

After the third immunization, cross-binding antibody titers to different S or RBD proteins increased by 1.1 to 40.6 times, and the significant increasements (>5-fold) in N501Y RBD-, Beta RBD-, and Delta RBD-binding antibody titers were detected among all the groups (Figure 4a). Notably, in addition to S1-2P-, S2-2P-, and S1-D614G-binding antibodies, high tiers of Omicron S trimer-targeting antibodies were detected in all groups (EC_50_ > 1 × 10^4^), and 5 μg S1-2P immunization elicited the highest antibody titer (EC_50_ > 4 × 10^4^). As for cross-binding antibody titers to different RBD proteins, the 20 μg S2-2P group induced significantly higher E484K RBD binding titer than that of the 10 μg dose group (*p* < 0.05), while the differences in antibody titers to other RBD proteins within a group or between different immune groups were not significant. Especially, the RBD-specific antibody titers elicited by the 2.5 μg S1-2P and 2.5 μg S2-2P bivalent vaccine groups were not inferior to other groups, indicating a low dose could work for a boost immunization. 

### 3.4. Cross-Neutralizing Antibody Responses to SARS-CoV-2 Variants

**Figure 5:**
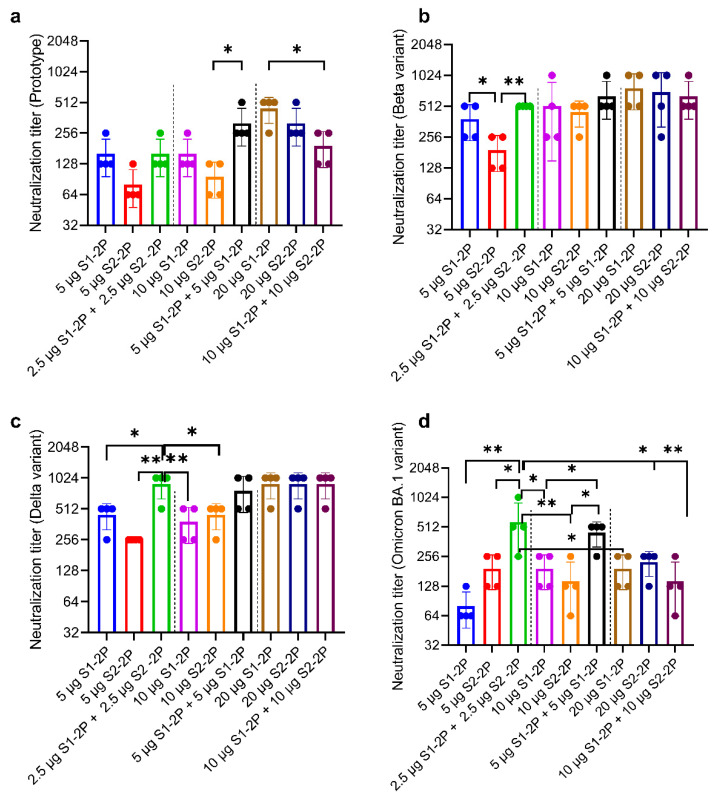
Analysis of neutralizing antibodies in the serum of vaccinated mice. Neutralization titers of vaccinated mouse sera against SARS-CoV-2 prototype (**a**), Beta variant (**b**), Delta variant (**c**), and Omicron variant (**d**). Live SARS-CoV-2 virus neutralization was accessed in sera of vaccinated mice collected two weeks after the third immunization. The dots of different colors represented mouse serum of different immune groups. The data were represented as the reciprocal of the serum dilution that protected Vero-E6 cells from CPE compared with no serum control. Statistically significant differences were compared using one-way ANOVA or *t*-test and are indicated as * for *p* < 0.05 and ** for *p* < 0.01.

The neutralizing activity against prototype, Beta, Delta, and Omicron variants of the immunized mice sera was then evaluated using a CPE assay. For groups immunized with S1-2P or S2-2P alone, the 20 μg group induced the highest neutralizing antibody titer against all four tested SARS-CoV-2 variants (Figure 5). For bivalent vaccine groups, a low dose (2.5 μg) could elicit neutralizing antibodies with titers higher than 128 (ranging from 128 to 1024). The bivalent vaccine containing S1-2P plus S2-2P at 2.5 μg elicited significantly higher neutralizing antibody titer than the 5 μg monovalent S2-2P vaccine (*p* < 0.05) against the Beta variant (Figure 5b) and significantly higher Delta variant-neutralizing antibody titer than monovalent vaccine formulated with 5 μg S1-2P (*p* < 0.05), 10 μg S1-2P (*p* < 0.01), 5 μg S2-2P (*p* < 0.01) and 10 μg S2-2P (*p* < 0.05) (Figure 5c), and also a significantly higher Omicron BA.1 variant-neutralizing antibody titer than other monovalent vaccine groups (Figure 5d). Especially, the neutralizing antibody titers against the prototype were not higher than the Beta variant and Delta variant, and the Omicron variant was the most difficult to be neutralized. Overall, either a high dose (20 μg) of monovalent S1-2P or S2-2P subunit vaccine or a low dose of bivalent vaccine S1-2P and S2-2P (2.5 μg for each) could induce a strong cross-neutralizing antibody response in mice. 

### 3.5. SARS-CoV-2 S-Specific Cellular Immune Responses

**Figure 6:**
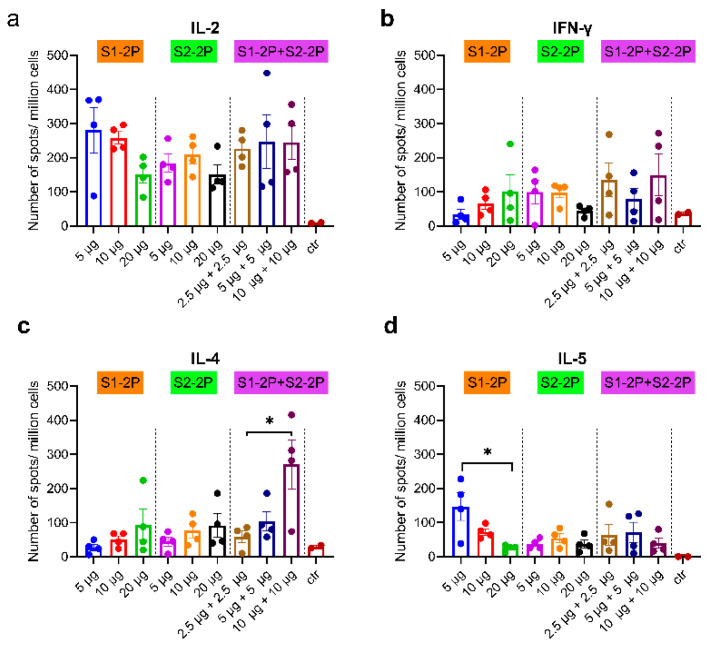
Spike-specific cellular immune responses. IL-2 (**a**), IFN-γ (**b**), Il-4 (**c**), and IL-5 (**d**) T cell responses of splenocytes from each mouse were shown as mean ± SEM for each animal. The dots of different colors represented mouse serum of different immune groups. Statistically significant differences were compared using one-way ANOVA or *t*-test and are indicated as * for *p* < 0.05.

To evaluate antigen-specific cell-mediated immunity (CMI) of the recombinant S trimer, splenocytes from the immunized mice were separated 14 days after the third vaccination, followed by stimulation with S trimer antigens and detection of Th1 (IL-2 and IFN-γ) and Th2 (IL-4 and IL-5) CMI with an ELISPOT assay. As shown in Figure 6, all three S trimer vaccine groups elicited robust T-cell responses against the antigen proteins (S1-2P and S2-2P), especially IL-2 responses. A Th1-biased cell-mediated immune response was observed in most groups, except that the bivalent vaccine with 10 μg S1-2P and 10 μg S2-2P induced a stronger IL-4 response than other groups, and 5 μg S1-2P monovalent vaccine induced a significant stronger IL-5 response than 20 μg S1-2P (*p* < 0.05). CMI appeared to be independent of the dose of S trimer used for vaccination. The bivalent vaccine with a low dose of 2.5 μg S1-2P and 2.5 μg S2-2P could elicit robust cellular immune responses.

## 4. Discussion

Different types of COVID-19 vaccines developed from the prototype SARS-CoV-2 have been approved or authorized for emergency use in humans. These vaccines provide efficient protection from severe infections; however, the newly emerged SARS-CoV-2 variants carry multiple mutations in key epitopes that can escape neutralizing responses elicited by prototype SARS-CoV-2 vaccines [20]. Thus, variants-based second-generation vaccines, either in monovalent or multivalent form that elicit broadly protective efficacy, should be developed to overcome the threat caused by the neutralizing escape of emerging variants. 

Among the multiple SARS-CoV-2 variants reported, the Beta variant (B.1.351) attracted much attention for reducing vaccine efficacy and escaping from the neutralization of antibodies in convalescent and vaccinator sera [19,30,31]. The Beta spike protein contained mutations on NTD and 3 typical mutations on RBD (K417N, E484K, and N501Y) that conferred higher affinity to ACE2 and neutralizing escape [32,33]. In this study, we first developed a monovalent vaccine with Beta spike trimer protein S1-2P adjuvanted with alum. We proved the vaccine-induced broad cross-binding antibody responses and cross-neutralizing activity to Beta, Delta, and Omicron variants. To enhance the cross-protection from other existing and future emerging variants, a spike trimer derived from B.1.618, which carries mutations of substitutions and deletions on NTD and E484K mutation on RBD, was also designed an S2-2P subunit vaccine and further formulated a bivalent vaccine with Beta spike trimer (S1-2P + S2-2P). As expected, the bivalent vaccine containing 2.5 μg S1-2P and S2-2P elicited a strong cross-neutralizing antibody response against Beta, Delta, and Omicron variants in mice, which was much higher than that induced by the monovalent vaccines, indicating the advantage of the bivalent vaccine in eliciting neutralizing antibody responses. In addition, at the same antigen dose, S1-2P induced higher titers of broadly neutralizing antibodies against prototype, Beta, Delta, and Omicron variants than that induced by S2-2P, indicating better immunogenicity of S1-2P. For both the monovalent and bivalent vaccine groups, the highest neutralizing titer against Beta and Delta variants reached 1024, which indicated a good potency for protection against Beta and Delta variants infection. It is not surprising that neutralizing titers against Omicron were low since Omicron was still the most difficult to neutralize among all the variants, and antibody evasion characters of the Omicron variant have been reported by recent studies [34,35,36]. However, the bivalent vaccine at 2.5 μg dose neutralized Omicron with an average titer of 512, indicating the potential to induce immune protection against Omicron. 

In this study, cross-binding antibody titers against S1-2P, S2-2P, S1-D614G protein, Omicron S trimer, and RBD proteins, including E484K, N501Y, Beta RBD, Gamma RBD, and Delta RBD in the immunized sera, were detected. Either the monovalent or the bivalent vaccine could potently induce a cross-variant neutralizing antibody response. Similar findings were reported for a bivalent S trimer vaccine SCTV01C that induced broad-spectrum cross-neutralizing activities against various SARS-CoV-2 variants [37]. Both S1-2P and S2-2P carry E484K mutation, and a dose-dependent effect was observed for E484K binding antibody levels in the monovalent S1-2P and monovalent S2-2P vaccine group after the second immunization. S1-2P vaccine-immunized group exhibited good cross-binding activity to the gamma RBD that might attribute to the similar mutation patterns of RBD with that of the Beta variant (K417N, E484K, N501Y) and Gamma variant (K417T, E484K, N501Y) [21]. Notably, the bivalent vaccine at 2.5 μg dose elicited significantly higher antibody responses towards N501Y RBD, Delta RBD, and Gamma RBD than other groups. The antibody responses were elevated after a second boost of the subunit vaccine in mice, as reported in [37]. Still, the dose effect observed in the study after the third immunization was not that obvious. One explanation could be the full activation of immune responses among groups after the third immunization that decreased the differences in antibody titers. Therefore, a low dose of S trimer protein could be adopted for boost immunization. 

In the immune response to defeat viral infection, other than humoral immunity, cellular immunity plays an important role in destroying infected cells and controlling the viral load in the body. With the continued emergence of new SARS-CoV-2 strains, it is urgently necessary to develop a universal vaccine that can induce both strong cellular and humoral immunity. It has been shown in a previous study [38] that the recombinant spike S trimer can induce Th1-biased protective immunity at a low dose. Immunizing S trimer with AS03 or CpG 1018 plus alum adjuvants induced Th1-biased CMI and high levels of neutralizing antibodies in animal models [26]. In this study, spleencytes isolated from S trimer immunized mice showed an increased production of IFN-γ, IL-2, IL-4, and IL-5 after stimulation, and the ratio of IFN-γ/IL-4 (higher than 1.0) indicated a Th1-biased cellular immunity. Moreover, the bivalent vaccine significantly improved the CMI responses at a low dose of 2.5 μg, which elicited a stronger Th1 immune response than the monovalent vaccine (*p* < 0.05). These data suggested that the S trimer vaccines, especially the bivalent vaccine with alum adjuvant, can induce not only efficient humoral responses but also cellular immune responses in mice. 

However, our current study has potential limitations. Firstly, adjuvants of different components could enhance the immune response to varying degrees through different mechanisms. In this study, the adjuvant used for the vaccine was restricted to two kinds (alum and an MF59-like adjuvant AddaVax). Our study showed that alum adjuvant induced better antibody responses than AddaVax, but other adjuvants, such as AS03 or CpG 1018, that might greatly enhance the immune responses, should also be considered in future studies [39]. Secondly, the lowest dose used in the study was 2.5 μg. Whether a lower dose (less than 2.5 μg) is also potent in eliciting broad-neutralizing antibody responses should be studied to optimize the dose of the vaccine.

In conclusion, our study demonstrated that SARS-CoV-2 S trimer strain-derived recombinant protein vaccines (monovalent or bivalent at a low dose) could not only induce robust cellular and humoral immune responses but also potently induce cross-variant neutralizing antibodies responses. These findings provide valuable information for developing variant-based monovalent or multivalent recombinant protein subunit SARS-CoV-2 vaccines.

## Data Availability

All data during the current study are available from the corresponding author on reasonable request.

## Supplementary Materials

The following supporting information can be downloaded at: https://www.mdpi.com/article/10.3390/vaccines11010193/s1, Table S1: Proteins used for ELISA.

## References

[1] Zhu N., Zhang D., Wang W., Li X., Yang B., Song J., Zhao X., Huang B., Shi W., Lu R. (2020). A Novel Coronavirus from Patients with Pneumonia in China, 2019. N. Engl. J. Med..

[2] Gao P., Liu J., Liu M. (2022). Effect of COVID-19 Vaccines on Reducing the Risk of Long COVID in the Real World: A Systematic Review and Meta-Analysis. Int. J. Environ. Res. Public Health.

[3] Peng Q., Zhou R., Wang Y., Zhao M., Liu N., Li S., Huang H., Yang D., Au K.K., Wang H. (2022). Waning immune responses against SARS-CoV-2 variants of concern among vaccinees in Hong Kong. EBioMedicine.

[4] Chen L.L., Lu L., Choi C.Y.K., Cai J.P., Tsoi H.W., Chu A.W.-H., Ip J.D., Chan W.-M., Zhang R.R., Zhang X. (2022). Impact of Severe Acute Respiratory Syndrome Coronavirus 2 (SARS-CoV-2) Variant-Associated Receptor Binding Domain (RBD) Mutations on the Susceptibility to Serum Antibodies Elicited by Coronavirus Disease 2019 (COVID-19) Infection or Vaccination. Clin. Infect. Dis..

[5] Wang R., Chen J., Wei G.W. (2021). Mechanisms of SARS-CoV-2 Evolution Revealing Vaccine-Resistant Mutations in Europe and America. J. Phys. Chem. Lett..

[6] Chemaitelly H., Tang P., Hasan M.R., AlMukdad S., Yassine H.M., Benslimane F.M., Al Khatib H.A., Coyle P., Ayoub H.H., Al Kanaani Z. (2021). Waning of BNT162b2 Vaccine Protection against SARS-CoV-2 Infection in Qatar. N. Engl. J. Med..

[7] Planas D., Bruel T., Grzelak L., Guivel-Benhassine F., Staropoli I., Porrot F., Planchais C., Buchrieser J., Rajah M.M., Bishop E. (2021). Sensitivity of infectious SARS-CoV-2 B.1.1.7 and B.1.351 variants to neutralizing antibodies. Nat. Med..

[8] Hoffmann M., Arora P., Groß R., Seidel A., Hörnich B.F., Hahn A.S., Krüger N., Graichen L., Hofmann-Winkler H., Kempf A. (2021). SARS-CoV-2 variants B.1.351 and P.1 escape from neutralizing antibodies. Cell.

[9] Planas D., Veyer D., Baidaliuk A., Staropoli I., Guivel-Benhassine F., Rajah M.M., Planchais C., Porrot F., Robillard N., Puech J. (2021). Reduced sensitivity of SARS-CoV-2 variant Delta to antibody neutralization. Nature.

[10] Perez-Then E., Lucas C., Monteiro V.S., Miric M., Brache V., Cochon L., Vogels C.B.F., Malik A.A., De la Cruz E., Jorge A. (2022). Neutralizing antibodies against the SARS-CoV-2 Delta and Omicron variants following heterologous CoronaVac plus BNT162b2 booster vaccination. Nat. Med..

[11] Tada T., Zhou H., Dcosta B., Samanovic M., Chivukula V., Herati R., Hubbard S., Mulligan M., Landau N. (2022). Increased resistance of SARS-CoV-2 Omicron variant to neutralization by vaccine-elicited and therapeutic antibodies. EBioMedicine.

[12] Suryawanshi R.K., Chen I., Ma T., Syed A., Brazer N., Saldhi P., Simoneau C., Ciling A., Khalid M., Sreekumar B. (2022). Limited cross-variant immunity from SARS-CoV-2 Omicron without vaccination. Nature.

[13] Walls A.C., Park Y.J., Tortorici M.A., Wall A., McGuire A.T., Veesler D. (2020). Structure, Function, and Antigenicity of the SARS-CoV-2 Spike Glycoprotein. Cell.

[14] Robbiani D.F., Gaebler C., Muecksch F., Lorenzi J., Wang Z., Cho A., Agudelo M., Barnes C., Gazumyan A., Finkin S. (2020). Convergent antibody responses to SARS-CoV-2 in convalescent individuals. Nature.

[15] Wang H., Yan D., Li Y., Gong Y., Mai Y., Li B., Zhu X., Wan X., Xie L., Jiang H. (2022). Clinical and antibody characteristics reveal diverse signatures of severe and non-severe SARS-CoV-2 patients. Infect. Dis. Poverty.

[16] Rolland M., Gilbert P.B. (2021). Sieve analysis to understand how SARS-CoV-2 diversity can impact vaccine protection. PLoS Pathog..

[17] Mohammadi M., Shayestehpour M., Mirzaei H. (2021). The impact of spike mutated variants of SARS-CoV2 [Alpha, Beta, Gamma, Delta, and Lambda] on the efficacy of subunit recombinant vaccines. Braz. J. Infect. Dis..

[18] Zhou D., Dejnirattisai W., Supasa P., Liu C., Mentzer A., Ginn H., Zhao Y., Duyvesteyn H., Tuekprakhon A., Nutalai R. (2021). Evidence of escape of SARS-CoV-2 variant B.1.351 from natural and vaccine-induced sera. Cell.

[19] Cele S., Africa N.F.G.S.I.S., Gazy I., Jackson L., Hwa S.-H., Tegally H., Lustig G., Giandhari J., Pillay S., Wilkinson E. (2021). Escape of SARS-CoV-2 501Y.V2 from neutralization by convalescent plasma. Nature.

[20] Lazarevic I., Pravica V., Miljanovic D., Cupic M. (2021). Immune Evasion of SARS-CoV-2 Emerging Variants: What Have We Learnt So Far?. Viruses.

[21] Moyo-Gwete T., Madzivhandila M., Makhado Z., Ayres F., Mhlanga D., Oosthuysen B., Lambson B., Kgagudi P., Tegally H., Iranzadeh A. (2021). Cross-Reactive Neutralizing Antibody Responses Elicited by SARS-CoV-2 501Y.V2 (B.1.351). N. Engl. J. Med..

[22] Ren W., Ju X., Gong M., Lan J., Yu Y., Long Q., Kenney D., O’Connell A., Zhang Y., Zhong J. (2022). Characterization of SARS-CoV-2 Variants B.1.617.1 (Kappa), B.1.617.2 (Delta), and B.1.618 by Cell Entry and Immune Evasion. MBio.

[23] Khan A., Gui J., Ahmad W., Haq I., Shahid M., Khan A.A., Shah A., Khan A., Ali L., Anwar Z. (2021). The SARS-CoV-2 B.1.618 variant slightly alters the spike RBD–ACE2 binding affinity and is an antibody escaping variant: A computational structural perspective. RSC Adv..

[24] Richmond P., Hatchuel L., Dong M., Ma B., Hu B., Smolenov I., Li P., Liang P., Han H.H., Liang J. (2021). Safety and immunogenicity of S-Trimer (SCB-2019), a protein subunit vaccine candidate for COVID-19 in healthy adults: A phase 1, randomised, double-blind, placebo-controlled trial. Lancet.

[25] Hsieh S.-M., Liu M.-C., Chen Y.-H., Lee W.-S., Hwang S.-J., Cheng S.-H., Ko W.-C., Hwang K.-P., Wang N.-C., Lee Y.-L. (2021). Safety and immunogenicity of CpG 1018 and aluminium hydroxide-adjuvanted SARS-CoV-2 S-2P protein vaccine MVC-COV1901: Interim results of a large-scale, double-blind, randomised, placebo-controlled phase 2 trial in Taiwan. Lancet Respir. Med..

[26] Liang J.G., Su D., Song T.-Z., Zeng Y., Huang W., Wu J., Xu R., Luo P., Yang X., Zhang X. (2021). S-Trimer, a COVID-19 subunit vaccine candidate, induces protective immunity in nonhuman primates. Nat. Commun..

[27] Li J.X., Zhu F.C. (2022). The S-Trimer (SCB-2019) COVID-19 vaccine and reinfection with SARS-CoV-2. Lancet Infect. Dis..

[28] Bravo L., Smolenov I., Han H.H., Li P., Hosain R., Rockhold F., Clemens S.A.C., Roa C., Borja-Tabora C., Quinsaat A. (2022). Efficacy of the adjuvanted subunit protein COVID-19 vaccine, SCB-2019: A phase 2 and 3 multicentre, double-blind, randomised, placebo-controlled trial. Lancet.

[29] Pino P., Kint J., Kiseljak D., Agnolon V., Corradin G., Kajava A., Rovero P., Dijkman R., den Hartog G., McLellan J. (2020). Trimeric SARS-CoV-2 Spike Proteins Produced from CHO Cells in Bioreactors Are High-Quality Antigens. Processes.

[30] Wibmer C.K., Ayres F., Hermanus T., Madzivhandila M., Kgagudi P., Oosthuysen B., Lambson B.E., De Oliveira T., Vermeulen M., Van der Berg K. (2021). SARS-CoV-2 501Y.V2 Escapes Neutralization by South African COVID-19 Donor Plasma. Nat. Med..

[31] Li Q., Nie J., Wu J., Zhang L., Ding R., Wang H., Zhang Y., Li T., Liu S., Zhang M. (2021). SARS-CoV-2 501Y.V2 variants lack higher infectivity but do have immune escape. Cell.

[32] Starr T.N., Greaney A., Hilton S., Ellis D., Crawford K., Dingens A., Navarro M., Bowen J., Tortorici M., Walls A. (2020). Deep Mutational Scanning of SARS-CoV-2 Receptor Binding Domain Reveals Constraints on Folding and ACE2 Binding. Cell.

[33] Fratev F. (2021). N501Y and K417N Mutations in the Spike Protein of SARS-CoV-2 Alter the Interactions with Both hACE2 and Human-Derived Antibody: A Free Energy of Perturbation Retrospective Study. J. Chem. Inf. Model..

[34] Cao Y., Wang J., Jian F., Xiao T., Song W., Yisimayi A., Huang W., Li Q., Wang P., An R. (2022). Omicron escapes the majority of existing SARS-CoV-2 neutralizing antibodies. Nature.

[35] Cele S., Jackson L., Khoury D., Khan K., Moyo-Gwete T., Tegally H., San J., Cromer D., Scheepers C., Amoako D. (2022). Omicron extensively but incompletely escapes Pfizer BNT162b2 neutralization. Nature.

[36] Liu L., Iketani S., Guo Y., Chan J., Wang M., Liu L., Luo Y., Chu H., Huang Y., Nair M. (2022). Striking antibody evasion manifested by the Omicron variant of SARS-CoV-2. Nature.

[37] Wang R., Sun C., Ma J., Yu C., Kong D., Chen M., Liu X., Zhao D., Gao S., Kou S. (2022). A Bivalent COVID-19 Vaccine Based on Alpha and Beta Variants Elicits Potent and Broad Immune Responses in Mice against SARS-CoV-2 Variants. Vaccines.

[38] Liao H.C., Wu W., Chiang C., Huang M., Shen K., Huang Y., Wu S., Liao C., Chen H., Liu S. (2022). Low-Dose SARS-CoV-2 S-Trimer with an Emulsion Adjuvant Induced Th1-Biased Protective Immunity. Int. J. Mol. Sci..

[39] Zhang N., Ji Q., Liu Z., Tang K., Xie Y., Li K., Zhou J., Li S., Shang H., Shi Z. (2022). Effect of Different Adjuvants on Immune Responses Elicited by Protein-Based Subunit Vaccines against SARS-CoV-2 and Its Delta Variant. Viruses.

