# Supplementary material for: S Trimer Derived from SARS-CoV-2 B.1.351 and B.1.618 Induced Effective Immune Response against Multiple SARS-CoV-2 Variants
Source: Vaccines (Basel). 2023 Jan 16;11(1):193. doi: 10.3390/vaccines11010193 (PMC9863711; doi:10.3390/vaccines11010193)
Supplement: Supplementary file 1 [file vaccines-11-00193-s001.zip › vaccines-2075661-supplementary.pdf]

Supplementary Table 1. Proteins used for ELISA

Name	Source	Identifier
S1-D614G	Novoprotein	Cat#DRA57
E484K RBD	Novoprotein	Cat#DRA134
N501Y RBD	Novoprotein	Cat#DRA120
Gamma RBD	Novoprotein	Cat#DRA144
Beta RBD	Novoprotein	Cat#DRA194
Delta RBD	SinoBiological	Cat#40592-V08H90
Omicron S trimer	Novoprotein	Cat#DRA125
